# Serum neurofilament light chain, contactin-1 and complement activation in anti-MAG IgM paraprotein-related peripheral neuropathy

**DOI:** 10.1007/s00415-022-10993-4

**Published:** 2022-02-14

**Authors:** Karima Amaador, Luuk Wieske, Marleen J. A. Koel-Simmelink, A. Kamp, Ilse Jongerius, Koen de Heer, Charlotte E. Teunissen, Monique C. Minnema, Nicolette C. Notermans, Filip Eftimov, Marie José Kersten, Josephine M. I. Vos

**Affiliations:** 1 Department of Hematology, Amsterdam UMC, University of Amsterdam, Cancer Center Amsterdam, LYMMCARE, Amsterdam, The Netherlands; 2 Department of Neurology and Neurophysiology, Amsterdam Neuroscience, Amsterdam UMC, Location AMC, Amsterdam, The Netherlands; 3 Neurochemistry Laboratory, Department of Clinical Chemistry, Amsterdam Neuroscience, Amsterdam UMC, Vrije Universiteit, Amsterdam UMC, Location VU Medical Center, Amsterdam, The Netherlands; 4 Department of Immunopathology, Sanquin Research and Landsteiner Laboratory, Amsterdam University Medical Centre, University of Amsterdam, Amsterdam, 1066CX The Netherlands; 5 Department of Pediatric Immunology, Rheumatology and Infectious Diseases, Emma Children’s Hospital, Amsterdam UMC, Amsterdam, The Netherlands; 6 Department of Internal Medicine, Flevo Hospital, Almere, The Netherlands; 7 Department of Hematology, University Medical Center Utrecht, University Utrecht, Utrecht, The Netherlands; 8 Department of Neurology, UMC Utrecht Brain Center, University Medical Center Utrecht, University Utrecht, Utrecht, The Netherlands

**Keywords:** Peripheral neuropathy, Anti-MAG neuropathy, IgM MGUS, Waldenstrom Macroglobulinemia, Biomarkers, Demyelinating diseases

## Abstract

**Introduction:**

In anti-myelin-associated glycoprotein IgM paraprotein-related peripheral neuropathy (anti-MAG PN), there is a lack of reliable biomarkers to select patients eligible for therapy and for evaluating treatment effects, both in routine practice and in clinical trials. Neurofilament light chain (NfL) and contactin-1 (CNTN1) can serve as markers of axonal and paranodal damage. Complement activation is involved in the pathogenesis in anti-MAG PN. We, therefore, hypothesized that serum NfL, CNTN1, C3b/c and C4b/c may function as biomarkers of disease activity in anti-MAG PN.

**Methods:**

In this prospective cohort study, we included 24 treatment-naïve patients with anti-MAG PN (mean age 69 years, 57% male) that had IgM paraproteinemia, a high IgM MAG-antibody, and clinical diagnosis of anti-MAG PN by a neurologist specialized in peripheral nerve disorders. We measured serum NfL, CNTN1, C3b/c and C4b/c, reference values were based on healthy controls. As controls, 10 treatment-naïve patients with IgM Monoclonal gammopathy of undetermined significance (MGUS) or Waldenström’s Macroglobulinemia (mean age 69 years, 60% male) without signs of neuropathy were included (non-PN).

**Results:**

NfL, CNTN1 levels in serum were mostly normal in anti-MAG PN patients and comparable to non-PN patients. C3b/c and C4b/c levels were normal in anti-MAG PN patients.

**Conclusion:**

Our results do not support serum NfL, CNTN1, and C3b/c and C4b/c as potential biomarkers in anti-MAG PN, although we cannot exclude that subgroups or subtle abnormalities could be found in a much larger cohort with longitudinal follow-up.

## Introduction

Anti-myelin-associated glycoprotein (MAG) IgM paraprotein-related peripheral neuropathy (anti-MAG PN) is the most prevalent variant of the immunoglobulin M (IgM) paraproteinemia-related peripheral neuropathies (PN) accounting for approximately 50% of cases [1]. It is a demyelinating neuropathy and typically presents as a chronic disorder with progressive imbalance, gait ataxia, sensory disturbances and tremor, leading to invalidating symptoms in up to 50% of patients [2]. Presence of IgM paraproteinemia and high titer anti-MAG antibodies are required for the diagnosis [3]. The underlying B-cell clone represents in most patients a premalignant state, i.e., IgM monoclonal gammopathy of undetermined significance (MGUS). In a minority of patients a diagnosis of Waldenström’s Macroglobulinemia (WM) can be made. Patients with anti-MAG PN often carry the somatic *MYD88*^*L265P*^ mutation [4, 5]. Treatment targeting the B-cell clone with the anti-CD20 antibody rituximab is recommended in severe and/or progressive cases, however, with only moderate success [6]. In addition, selecting candidates for treatment, tracking progression over time and monitoring response is difficult, because there is no reliable clinical score or biomarker to measure disease activity or clinical outcomes [7]. For example, anti-MAG titers and paraprotein levels do not correlate well with disease activity [8]. In the era of new therapeutic options for B-cell malignancies, such as oral Bruton tyrosine kinase (BTK) inhibitors, less neurotoxic second generation proteasome inhibitors, and highly effective complement inhibitors, reliable response biomarkers are vital for successful evaluation of treatment effects in the context of clinical trials [9, 10].

Anti-MAG antibodies are central in the pathogenesis causing complement mediated demyelination and nerve damage [11]. Proteins in blood reflecting nerve damage are increasingly used as biomarkers of disease activity in other PN. Neurofilament light chain (NfL), an axonal structural protein, is an established biomarker for axonal damage and was shown to be a response biomarker in hereditary transthyretin-mediated (hATTR) polyneuropathy and vasculitis neuropathy, and in selected cases, for chronic inflammatory demyelinating polyneuropathy (CIDP) [12–15]. So far, serum NfL has only been studied in 3 anti-MAG PN patients [16]. More recently, contactin-1 (CNTN1) has been identified as a potential biomarker for damage to the paranode (a nerve region in between the node of Ranvier and compact myelin) [17]. Paranodal alternations are a frequent and early pathological finding in anti-MAG PN [18]. The complement system is known to be involved in anti-MAG PN based on nerve biopsy studies. When IgM antibodies bind MAG, complement mediated demyelination occurs and deposits of IgM and complement can be observed in myelin sheets [11]. Serum CNTN1 and complement activation markers have not been studied yet in anti-MAG PN. We hypothesized that serum NfL, CNTN1 and complement activation product C3b/c and C4b/c levels are abnormal in anti-MAG PN compared to healthy donors and IgM MGUS/WM patients without PN, and that they are possible biomarkers for disease activity.

## Methods

We included treatment-naïve patients with anti-MAG PN (PN) who fulfilled the following criteria: IgM paraproteinemia, high titer MAG-antibodies (95% had > 10.000 Bühlmann titer units (BTU)), a clinical diagnosis of anti-MAG PN by a neurologist with expertise in peripheral nerve disorders confirmed by electromyography (EMG). The control group consisted of treatment-naïve patients with IgM MGUS or WM without clinical signs of neuropathy (non-PN). The non-PN group was included to allow for the ability to relate possible differences to the underlying B-cell clone. Established healthy control reference values for serum Nfl, CNTN1 and c3b/c and c4b/c were used for comparison as described below. Eligible patients were identified in the tertiary referral centers for inflammatory neuropathies and WM of the Amsterdam UMC and the University Medical Center Utrecht. Demographic data and clinical characteristics were recorded at inclusion. The Medical Ethical Committee of University Medical Center Utrecht (ID: 20/609) approved this study and all patients provided signed informed consent.

Sampling of peripheral blood took place before initiation of treatment. Centrifugation (1800 g, 10 min at room temperature) was performed within two hours and serum samples were stored at -80 °C. Serum NfL levels were measured using a highly sensitive single molecule array (Simoa) assay in the Neurochemistry Laboratory at Amsterdam UMC as previously described [12]. Serum CNTN1 levels were measured on Bio-Plex 200 system (Bio-Rad Laboratories, Veenendaal, The Netherlands) using the Human Magnetic Luminex Assay (LXSAHM; R&D Systems, Minneapolis, MN) according to the manufacturer's instructions, as described previously [17].

For NfL and CNTN1 data from 222 healthy controls (HCs) (mean age 46 years; SD 14; range 19–98 years) were used as lab-established reference values [12]. Abnormal NfL levels were defined as at or above the 95th percentile of age-specific cutoff values in HCs [12]. As CNTN1 does not vary with age, abnormal CNTN1 levels were defined as below the 5th percentile of values in HCs (appendix 1) [17]. Complement activation products of C3 (C3b, C3bi and C3c) and C4 (C4b, C4bi, C4c), collectively referred to as C3b/c and C4b/c, respectively, were measured in serum of a subgroup of PN patients by enzyme-linked immunosorbent assay (ELISA) as described previously [19]. Normal reference values were measured in a pool of HCs as well as individual donors and maximum C3b/c and C4b/c levels in HCs were used as cutoff values.

We used a two-way ANOVA test to investigate differences in (logarithmically transformed) NfL levels between groups corrected for age. Differences in CNTN1 levels between PN and non-PN groups were assessed with the Mann–Whitney *U* test. Data were analyzed using R, version 3.6.2.

## Results

**Fig. 1:**
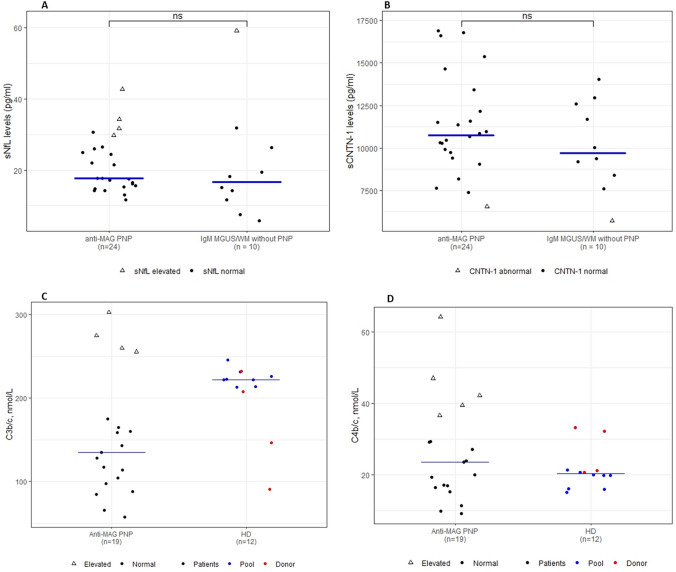
**A** Serum NfL in anti-MAG PN patients, and in IgM MGUS/WM patients without PNP. The median is represented by the blue horizontal bars. Closed circles represent normal serum NfL values (i.e., below the 95th percentile of age-specific cutoff values). Open triangles represent elevated serum NfL values (i.e., at or above the 95th percentile of age-specific cutoff values). **B** Serum Contactin-1 (CNTN1) Levels in anti-MAG PNP patients, and IgM MGUS/WM patients without PNP. Closed circles represent normal serum CNTN1 values. Open triangles represent abnormal serum CNTN1 values (i.e., below the 5th percentile of the HCs reference values). **C** Serum **A** C3b/c and **D** C4b/c concentrations in patients with anti-MAG PNP compared to HDs. HD data obtained from a pool are designated in blue and HD obtained from individual donors are designated in red. The lower median C3b/c levels in patients was not considered clinically relevant and is attributed to sample processing. The median is represented by the horizontal bars

A total of 24 PN patients and 10 non-PN controls were included. Patient characteristics are presented in Table 1. Nearly all anti-MAG PN patients (96%) presented with the classic phenotype of a chronic and slowly progressive sensorimotor neuropathy with the exception of one patient who presented with a subacute phenotype with rapid progression. When corrected for age, serum NfL levels did not differ between PN and non-PN patients (*P* = 0.3). Abnormal serum NfL levels were present in 4 (17%) PN patients (range serum NfL: 29.7–42.8 pg/mL) and in 1 (10%; 59.2 pg/mL) non-PN patient (Fig. 1A). Elevated serum NfL levels could not be explained by age, gender, clinical phenotype (including an acuter phenotype) and disease duration. Median serum CNTN1 levels were also similar between PN and non-PN patients (*P* = 0.4) (Table 1); 1 (4%) patient in the PN cohort and 1 (10%) patient in the non-PN cohort had abnormal CNTN1 levels (Fig. 1B). Median levels and interquartile range (IQR) of C3b/c, and C4b/c are presented in Table 1 and Fig. 1C, D. Compared to HCs, modestly increased levels of C3b/c and C4b/c were present in 4 (21%) and 5 (26%) patients with anti-MAG PN, respectively.

**Table 1:** Clinical characteristics, serum neurofilament light chain levels (NFL), serum contactin-1 (CNTN1) levels, and complement activation of patients with IgM anti-MAG peripheral neuropathy and IgM MGUS/WM without PN

	Patients with IgM anti-MAG PN (*n* = 24)	IgM MGUS/WM patients without PN (*n* = 10)	*P* value
Age (years), mean (range)	69 (55–80)	69 (44–81)	–
Male, *n* (%)	13 (57%)	6 (60%)	–
Duration of disease (years, range)	4.5 (0–13)	–	–
Underlying hematologic diagnosis			
IgM MGUS	20 (87%)	6 (60%)	–
WM	3 (13%)	4 (40%)	–
Serum IgM (mg/dL)	670 (170–2940)	1912 (494–5514)^a^	–
Anti-MAG titer > 10.000, *n* (%)	20 (95%)	–	–
Presence of monoclonal cells in the bone marrow (No. (%) patients)	7 (39%)^b^	5 (83%)^c^	–
Serum NfL, median (IQR)	17.7 (15.5–26.1)	16.6 (12.3–24.5)	0.42
Abnormal serum NfL, *N* (%)	4 (17%)	1 (10%)	N.A
Abnormal serum NfL level range	29.7–42.8	59.2	
Serum CNTN1, median (IQR)	10,761 (9658–12,492)	9708 (8600–12,373)	0.38
Abnormal serum CNTN1, *N* (%)	1 (4%)	1 (10%)	N.A

	Patients with IgM anti-MAG PN (*n* = 19)	Healthy controls (*n* = 12)	*P* value
C3b/c (mmol/L), median (IQR)	135 (101–170)	222 (212–227)	
abnormal C3b/c, *N* (%)	4 (21%)	–	–
abnormal C3b/c level range	256–303	–	–
C4b/c (mmol/L), median (IQR)	23.5 (16.7–33)	20.3 (18.9–21.2)	
Abnormal C4b/c, *N* (%)	5 (26%)	–	–
Abnormal C4b/c range	36.6–64.2	–	–

## Discussion

In the current study, we found no significant differences in serum NfL and serum CNTN1 levels between anti-MAG PN and non-PN patients and levels for these biomarkers were mostly within normal ranges. C3b/c and C4b/c levels were also within normal ranges in the majority of PN patients.

Besides demyelination, axonal and paranodal damage can be found in nerve biopsies of anti-MAG patients, especially in the case of a longer disease course [18]. The normal NfL and CNTN1 levels we observed might be explained by the possibility that these processes might occur at a too slow pace to be captured by these biomarkers cross-sectionally. In other demyelinating polyneuropathies with an acute or subacute presentation, such as Guillain–Barre syndrome or acute-onset CIDP, abnormal levels of NfL can be frequently observed [12, 20]. Interestingly, however, one anti-MAG PN patient with a rapidly progressive and severe presentation also had normal levels for serum NfL and CNTN1. In four patients with elevated NfL levels, CNTN1 levels were normal and only two of these four patients had elevated C3b/c and C4b/c levels with a classic phenotype at presentation. We did not find differences in systemic complement activation between PN patients and HCs. The normal serum complement levels in the current study might be explained by the very local activation of complement in nerve fibers only, that does not reach the threshold for elevated serum levels [21].

Our data show that these biomarkers are not suitable for distinguishing anti-MAG PN patients from healthy donors or from WM/IgM MGUS patients without PN. Our study is limited by the small sample size. Therefore, we cannot rule out the possibility that subtle differences or subgroups with biochemically active disease (that can be identified using a combination of these biomarkers) might be observed in a much larger cohort. If such a much larger study were to be done, it might also be interesting to investigate changes in these biomarkers within an individual longitudinally. Such a study may be challenging due to the rare nature of anti-MAG PN and the lack of a clinical “gold standard” for disease activity. Indeed, this is also a limitation in our study. Nerve damage biomarkers specific for damage to compact myelin could be an alternative; however, these are currently unavailable and the pace of nerve damage might again be too slow [7]. Pathomechanistic biomarkers such as sensitive measurements of the IgM paraprotein or the anti-MAG antibodies themselves may be more responsive as suggested by a recent meta-analysis [22].

We conclude that serum NfL, CNTN1 and C3/C4 levels are normal in the majority of anti-MAG PN patients and, therefore, do not seem suited as biomarkers in clinical practice. However, we cannot rule out that investigation in a much larger cohort with longitudinal follow-up could identify subgroups that would benefit from these markers.

## References

[1] Svahn J, Petiot P, Antoine JC, Vial C, Delmont E, Viala K (2018). Anti-MAG antibodies in 202 patients: clinicopathological and therapeutic features. J Neurol Neurosurg Psychiatry.

[2] Smith IS (1994). The natural history of chronic demyelinating neuropathy associated with benign IgM paraproteinaemia. A clinical and neurophysiological study. Brain.

[3] Latov N (2021). Antibody testing in neuropathy associated with anti-Myelin-Associated Glycoprotein antibodies: where we are after 40 years. Curr Opin Neurol.

[4] Vos JM, Notermans NC, D'Sa S, Lunn MP, van der Pol WL, Kraan W (2018). High prevalence of the MYD88 L265P mutation in IgM anti-MAG paraprotein-associated peripheral neuropathy. J Neurol Neurosurg Psychiatry.

[5] Nobile-Orazio E, Gallia F, Terenghi F, Allaria S, Giannotta C, Carpo M (2008). How useful are anti-neural IgM antibodies in the diagnosis of chronic immune-mediated neuropathies?. J Neurol Sci.

[6] Galassi G, Tondelli M, Ariatti A, Benuzzi F, Nichelli P, Valzania F (2017). Long-term disability and prognostic factors in polyneuropathy associated with anti-myelin-associated glycoprotein (MAG) antibodies. Int J Neurosci.

[7] Wieske L, Smyth D, Lunn MP, Eftimov F, Teunissen CE (2021). Fluid biomarkers for monitoring structural changes in polyneuropathies: their use in clinical practice and trials. Neurotherapeutics.

[8] Pruppers MHJ, Merkies ISJ, Lunn MPT, Notermans NC (2017). 230th ENMC International Workshop: Improving future assessment and research in IgM anti-MAG peripheral neuropathy: a consensus collaborative effort, Naarden, The Netherlands, 24–26 February 2017. Neuromuscul Disord.

[9] Treon SP, Tripsas CK, Meid K, Warren D, Varma G, Green R (2015). Ibrutinib in previously treated waldenström’s macroglobulinemia. N Engl J Med.

[10] Kersten MJ, Amaador K, Minnema MC, Vos JMI, Nasserinejad K, Kap M (2021). Combining ixazomib with subcutaneous rituximab and dexamethasone in relapsed or refractory waldenström's macroglobulinemia: final analysis of the phase I/II HOVON124/ECWM-R2 study. J Clin Oncol.

[11] Briani C, Ferrari S, Campagnolo M, Tagliapietra M, Castellani F, Salvalaggio A (2021). Mechanisms of nerve damage in neuropathies associated with hematological diseases: lesson from nerve biopsies. Brain Sci.

[12] van Lieverloo GGA, Wieske L, Verhamme C, Vrancken AFJ, van Doorn PA, Michalak Z (2019). Serum neurofilament light chain in chronic inflammatory demyelinating polyneuropathy. J Peripher Nerv Syst.

[13] Bischof A, Manigold T, Barro C, Heijnen I, Berger CT, Derfuss T (2018). Serum neurofilament light chain: a biomarker of neuronal injury in vasculitic neuropathy. Ann Rheum Dis.

[14] Nioi P, Ticau S, Sridharan G, Tsour S, Cantley W, Chan A (2020). Neurofilament light chain (NfL) as a potential biomarker in hereditary transthyretin-mediated (hATTR) amyloidosis (771). Neurology.

[15] Khalil M, Teunissen CE, Otto M, Piehl F, Sormani MP, Gattringer T (2018). Neurofilaments as biomarkers in neurological disorders. Nat Rev Neurol.

[16] Mariotto S, Farinazzo A, Magliozzi R, Alberti D, Monaco S, Ferrari S (2018). Serum and cerebrospinal neurofilament light chain levels in patients with acquired peripheral neuropathies. J Peripher Nerv Syst.

[17] Wieske L, Martín-Aguilar L, Fehmi J, Lleixà C, Koel-Simmelink MJA, Chatterjee M (2021). Serum contactin-1 in CIDP. Neurol Neuroimmunol Neuroinflamm.

[18] Kawagashira Y, Koike H, Tomita M, Morozumi S, Iijima M, Nakamura T (2010). Morphological progression of myelin abnormalities in IgM-monoclonal gammopathy of undetermined significance anti-myelin-associated glycoprotein neuropathy. J Neuropathol Exp Neurol.

[19] Emmons TR, Giridharan T, Singel KL, Khan ANH, Ricciuti J, Howard K (2021). Mechanisms driving neutrophil-induced T-cell immunoparalysis in ovarian cancer. Cancer Immunol Res.

[20] Altmann P, De Simoni D, Kaider A, Ludwig B, Rath J, Leutmezer F (2020). Increased serum neurofilament light chain concentration indicates poor outcome in Guillain-Barré syndrome. J Neuroinflamm.

[21] De Letter MA, van Doorn PA, Savelkoul HF, Laman JD, Schmitz PI, de Coul AA (2000). Critical illness polyneuropathy and myopathy (CIPNM): evidence for local immune activation by cytokine-expression in the muscle tissue. J Neuroimmunol.

[22] Hänggi P, Aliu B, Martin K, Herrendorff R, Steck AJ (2022). Decrease in serum anti-MAG autoantibodies is associated with therapy response in patients with anti-MAG neuropathy. Neurol Neuroimmunol Neuroinflamm.

